# From early methods for DNA diagnostics to genomes and epigenomes at high resolution during four decades – a personal perspective

**DOI:** 10.48101/ujms.v129.11134

**Published:** 2024-12-09

**Authors:** Ann-Christine Syvänen

**Affiliations:** Department of Medical Sciences, Molecular Medicine and Science for Life Laboratory, Uppsala University, Uppsala, Sweden

**Keywords:** Genomics, genetics, SNP genotyping, polymerase chain reaction, microarrays, next generation sequencing, epigenomics, systemic lupus erythematosus, acute lymphoblastic leukemia, Finnish disease heritage, whole genome sequencing, single cell transcriptomics

## Abstract

In the 1980s, my research career begun with microbial DNA diagnostics at Orion Pharmaceutica in Helsinki, Finland, where I was part of an innovative team that developed novel methods based on the polymerase chain reaction (PCR) and the biotin–avidin interaction. One of our key achievements during this time was the invention of the solid-phase minisequencing method for genotyping single nucleotide polymorphisms (SNPs). In the 1990s, I shifted focus to human genetics, investigating mutations of the ‘Finnish disease heritage’. During this period, I also developed quantitative methods using PCR and minisequencing of mitochondrial mutations and for forensic analyses. In the late 1990s and early 2000s, microarray-based SNP genotyping became a major topic for my research, first in Helsinki and later with my research group at Uppsala University in Sweden. By the mid-2000s, I began collaborating with leading clinicians on genetics of autoimmune disease, specifically systemic lupus erythematosus and later worked on the classification and clinical outcome of pediatric acute lymphoblastic leukemia, when large-scale genomics and epigenomics emerged. These collaborations, which focused on integrating genomics into clinical practice, lasted almost two decades until I retired from research in 2022. In parallel with my research activities, I led the SNP/DNA Technology Platform in the Wallenberg Consortium North program from 2001 to 2006. I continued as Director of the SNP&SEQ Technology Platform, which expanded rapidly during the 2010s, and became part of Science for Life Laboratory in 2013. Today (in 2024), the SNP&SEQ Technology Platform is one of the largest units of the Swedish National Genomics Infrastructure hosted by SciLifeLab. The present article provides a personal perspective on nearly four decades of research, highlighting projects and methods I found particularly exciting or important.

**Figure:**
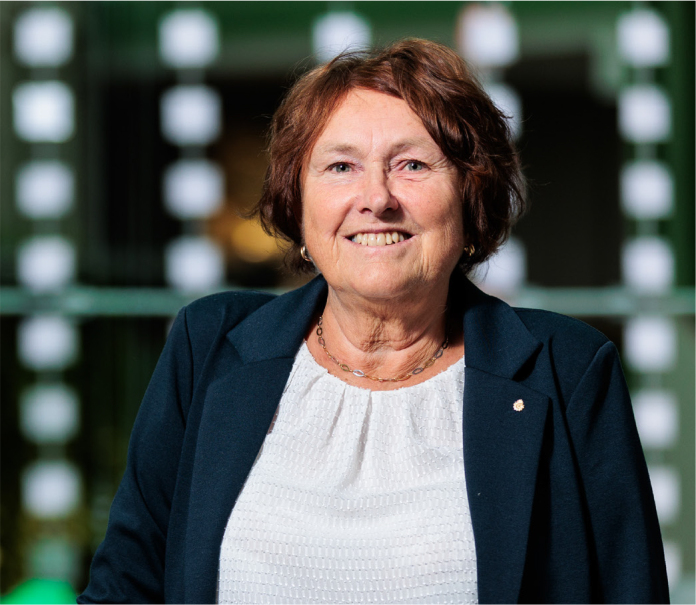
Professor Ann-Christine Syvänen, winner of the Medical Faculty of Uppsala University Rudbeck Award 2023. Photo: Tobias Sterner.

## Early methods for DNA diagnostics of infectious diseases

In 1983, I was fortunate to be recruited to the newly established Gene Technology Laboratory at Orion Pharmaceutica in Helsinki, Finland, to take part in the development of DNA hybridization methods for diagnosis of infectious diseases. I was recruited to this lab by Drs Hans Söderlund and Marjut Ranki at the Department of Virology at the University of Helsinki. The new Gene Technology Laboratory was located in an academic environment, in the same building as the Institute of Biotechnology at the University of Helsinki.

### Sandwich hybridization

**Figure 1:**
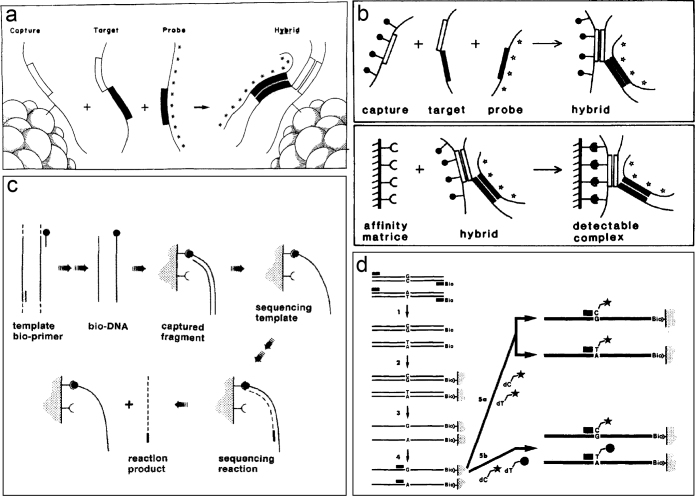
Principles of four early DNA-based methods: (a) sandwich hybridization, (b) affinity-based hybrid collection, (c) direct solid-phase sequencing of PCR products, (d) solid-phase minisequencing of PCR products.

As DNA-based diagnostics became increasingly important in clinical settings, there was a need for more practical and efficient nucleic acid hybridization assays than those originally developed in the 1960s for gene or organism identification in basic research (1). Marjut Ranki and Hans Söderlund had developed a ‘sandwich hybridization’ assay (Figure 1a), which was a promising tool for DNA-based routine microbial diagnostics (2). In the sandwich hybridization, a pair of non-overlapping DNA probes cloned from the target microbial DNA was used in the assay. In the initial assay version, a capturing DNA probe was immobilized on a small nitrocellulose filter disk, and a second probe was radioactively labeled with iodine-125. The microbial target DNA was allowed to hybridize to these two probes in solution essentially without purification from the crude biological material, followed by measurement of the formed ‘sandwich hybrid’ in a scintillation counter. The method was applied to the diagnosis and typing of adenovirus (3) and cytomegalovirus (CMV) (4) and later to several other viruses and bacteria, e.g. human papilloma virus, human immunodeficiency virus, hepatitis B, chlamydia, and E. coli strains.

To improve the sensitivity of sandwich hybridization and to use non-radioactive labeling for routine clinical diagnostics, we explored time resolved fluorometry with Europium (Eu) lanthanide chelates as label, developed by Wallac OY in Turku, Finland (5, 6). A labeled probe DNA was tagged with a hapten in a two-step immunochemical assay, in which the secondary antibody was labeled with an Eu-chelate. The target DNA was hybridized to the Eu-labeled probe in solution and to a capturing probe immobilized on small nitrocellulose filters placed in microtiter plate wells. After hybridization, the Eu-chelate was excited with a light pulse, and the photons from the long-lived fluorescence of the Eu-chelate were detected after a time delay, which suppressed the short-lived background fluorescence, increasing signal-to-noise ratio. The detection sensitivity of this quantitative assay was 10-fold higher than that of colorimetric enzyme immunoassays under the same conditions.

### Affinity-based hybrid collection

We also devised an affinity-based sandwich hybridization assay, in which a capturing DNA probe carrying biotin residues was used as affinity label for the collection of the DNA sandwich hybrids on streptavidin-coated agarose beads. After hybridization with the microbial target DNA in solution, a second labeled DNA probe carrying 125-iodine was used for quantification of the collected DNA hybrids (Figure 1b) (7). The major advantages of affinity-based sandwich hybridization in solution were fast reaction kinetics, high specificity when using two non-overlapping probes, and high sensitivity also in clinical samples containing crude biological material. The detection sensitivity of affinity-based sandwich hybridization was appropriate for special clinical cases, like CMV infections in newborns, but not for comprehensive diagnostics of microbial specimen without prior enrichment.

## The polymerase chain reaction

In the early 1980s, Kary Mullis invented the polymerase chain reaction (PCR), a method that in a simple manner allows for virtually unlimited exponential amplification of any DNA fragment with the aid of a pair of oligonucleotide primers and a DNA polymerase (8, 9). The only prerequisite for PCR is that the nucleotide sequence of a gene, gene region, or primer-binding fragment of interest is known. PCR made it possible to design convenient assays based on oligonucleotide primers instead of cloned DNA fragments, which were less practical due to the labor-intensive cloning processes and challenges in maintaining and reproducing cloned material. Kary Mullis received the Nobel Prize in Chemistry in 1993 for the invention of the PCR method.

In our laboratory, we immediately realized that PCR could remove the sensitivity issues in the diagnosis of infectious diseases. We also recognized that PCR could provide the sensitivity and specificity required for direct detection of single nucleotide variations in the human genome. We utilized PCR to devise a new version of the affinity-based hybrid collection method, in which we used PCR with biotinylated primers and a labeled probe to quantify PCR products. Affinity-based hybrid collection became a valuable tool to explore the properties of PCR (10). We learned that PCR is extremely sensitive to DNA contaminations, and that the efficiency of the reaction depends strongly on the concentration of the DNA template. This ‘plateau effect’ of PCR ruled out direct quantitative analysis of the amount of DNA that was initially subjected to amplification without an internal standard. I was the first person in Finland to perform PCR experiments. Initially, we performed PCR manually in Eppendorf tubes in a heat block. There was no thermoresistant DNA polymerase, so we had to open the test tubes and add the DNA polymerase at each PCR cycle, at a high risk for contaminating DNA. In 1988, our laboratory acquired the first ‘automated’ PCR machine and thermoresistant DNA polymerase became available, so we were able to scale up the experiments.

PCR was later adapted to a variety of innovative assay formats in thousands of laboratories and has served as an irreplaceable tool for diagnosis of infectious diseases and human genetic variation. Much later during 2002–2005, PCR was crucial for the construction of a map of linked nucleotide variations (haploptypes) across the human genome, which enabled genome-wide disease association studies (11), and more recently, PCR played a key role for diagnosis and management of COVID-19 in millions of patients across the world during the pandemic in 2020–2022 (12).

## Solid-phase sequencing of human genes

Using the biotin-avidin affinity capture principle described earlier, we devised a PCR-based method with solid-phase sequencing as read-out (13). We applied the method to the detection of two single nucleotide polymorphisms (SNPs) in the human Apolipoprotein E gene (ApoE), which had been shown by isoelectric focusing on the protein level to correspond to six possible combinations of three polymorphic ApoE alleles (14). The ApoE polymorphisms are clinically relevant for lipoprotein metabolism and cholesterol transport (15). The ApoE4 allele is today recognized as a well-established genetic risk factor for Alzheimer’s disease (16).

We performed PCR to amplify a 265 base pair fragment of the ApoE gene in six patients using a 5’-biotinylated and an unbiotinylated primer, captured the biotinylated PCR product on avidin-coated microparticles, and removed the unbiotinylated strand by alkaline denaturation (Figure 1c). The immobilized single-stranded PCR product was sequenced by the dideoxy-chain termination method using radioactive (^32^P) labeling (17), followed by size separation and analysis of the sequence reads on a polyacrylamide gel after autoradiography. We were able to successfully detect the alleles of two polymorphic nucleotide positions of the ApoE gene in homozygous or heterozygous form in six analyzed patients.

In collaboration with Thomas Hultman and Mathias Uhlén (KTH Royal Institute of Technology), we explored automation of the solid-phase sequencing method for genotyping of ApoE. Here, we used fluorescently labeled nucleotides and analyzed the sequenced fragments using an early version of the automated laser fluorescent sequencer from Pharmacia LKB (18).

## Solid-phase ‘minisequencing’ of single nucleotide variants

Solid-phase sequencing of affinity-captured PCR products amplified from human genes is an accurate method for the detection of human genetic variants. However, when the disease-causing mutations or risk variants of a gene are known, complete sequencing of PCR amplified fragments produces a large amount of redundant sequence information. This led us to devise the solid-phase minisequencing method for genotyping single nucleotide variants (SNPs) (19). In 1990, we submitted a patent application for a ‘Method for determining specific nucleotide variations in the presence of mixture of labeled nucleotides and terminators’, which was approved 10 years later after a long legal process with two other groups with similar inventions (Söderlund & Syvänen, 1991, PCT publication number WO 91/13075). The patent was accepted in Europe and USA, as European Patent No 0648280 Issued May 12^th^ 1999 and US patent No 6,013,431 Issued Jan. 11^th^ 2000. The patent expired in 2018.

As proof-of-principle for solid-phase minisequencing, we genotyped the two polymorphic ApoE variants in genomic DNA from the six patient samples described earlier. In the first data, there was a 100-fold signal-to-noise ratio between the allelic variants of ApoE, and the correct genotypes for each of the six samples were assigned. This immediately told me that the method would become a valuable genotyping tool! Here, we used labeling with ^135^S[dNTP] in two separate reactions, or alternatively, dual labeling with ^32^P[dNTP] and ^3^H[dNTP] in a single reaction mixture (Figure 1d). For non-radioactive labeling, haptens could be incorporated into DNA by solid-phase minisequencing, followed by colorimetric detection by an antibody-alkaline phosphatase conjugate (20). The optimal non-radioactive labeling method for solid-phase minisequencing eventually involved fluorescent dideoxy-nucleotides (ddNTPs), which became available in the early 2000s (New England Nuclear, NEN).

The ApoE gene is highly guanidine (G)- and cytosine (C)-rich, making it a challenging target to amplify by PCR. The fact that solid-phase minisequencing of ApoE was highly specific and sensitive convinced us that this method would provide a universal tool for genotyping all types of single-nucleotide variants (SNVs). I consider the invention of the solid-phase minisequencing method as the greatest achievement of my scientific career. Our first publication of the method has over 300 citations (21), and our development of the method has inspired many groups to devise and use genotyping assays based on single-nucleotide primer extension.

## Detection of disease-causing mutations in the human genome

In 1990, I applied for a position at the National Public Health Institute in the Department of Human Molecular Genetics headed by Dr Leena Peltonen. She seemed enthusiastic by my application and immediately offered me a position as a senior scientist in her laboratory, an arrangement of mutual benefit. I wanted to gain competence in human genetics, and Leena Peltonen was eager to establish the PCR technique, which I had experience from, as fast as possible in her lab.

### Molecular genetics of the Finnish disease heritage

A major interest in Leena Peltonen’s laboratory was to identify genes and mutations of the so-called Finnish disease heritage, comprising some 36 rare mostly recessively inherited monogenic diseases (22). The Finnish disease heritage was due to a population bottleneck among ancestors of contemporary Finns about 4,000 years ago (23). Consequently, its genetic variants are much more common in descendants of ethnic Finns than in other populations. When I joined Lena Peltonen’s laboratory in 1990, her group was busy searching for disease genes and their mutations by positional cloning methods. At this early time for genomics, little was known about the structure and sequence of the human genome. Notably, the Human Genome Project that aimed at sequencing the complete human genome was initiated in 1990, and the first draft sequence of the genome was published 13 years later, in 2003.

### Aspartylglucosaminuria

In 1990, Leena Peltonen’s PhD student Elina Ikonen had just isolated a complementary DNA (cDNA) that encodes the human aspartylglucosamindase (AGA) enzyme from a fetal liver cDNA library. By sequencing cDNA from an Aspartylglucosaminuria (AGU) patient using PCR, Elina Ikonen identified a missense mutation involving a G_163_ > C transition, which resulted in the substitution of a cystein residue with serine. This alteration disrupted the AGA protein and led to loss of AGA activity (24), and loss of AGA activity triggered the AGU disease. The main clinical manifestation of AGU is progressive intellectual disability of children from the age 2-5 years (25). AGA was the first gene from the Finnish disease heritage to be cloned and in which the disease-causing effect of the mutation was functionally verified.

My contribution to the AGU project was to determine the prevalence of the major AGU mutation in Finland (26). By analyzing DNA samples from 115 Finnish AGU patients, 57 family members, and 120 unrelated controls, we found that 98% of the AGU alleles in this population sample carried the same G_163_ > C transition in the AGA polypeptide. We identified only five apparently compound heterozygote patients with the major AGU mutation on one allele, and later these five alternative AGU mutations were identified in the AGU gene on the other allele (27). Among the control individuals, four carriers of the major AGU allele were identified, which corresponds to a carrier frequency of 1:30 in the analyzed population samples.

### Quantitative PCR of nucleotide sequence variants

Using PCR and solid-phase minisequencing, we devised a method for quantitative genotyping of mutations or sequence variants in DNA or RNA (cDNA) using ^3^H[dNTPs] as label. Accurate quantitative genotyping by PCR is feasible for two sequences that are highly similar, which are amplified with the same efficiency. The simultaneous amplification of two alleles of a mutant gene, which are identical except at the single mutant position, provides ideal internal controls for each other during PCR. The result of a minisequencing reaction is a ratio (R) between two incorporated ^3^H[dNTPs] that represent the relative amount of the two alleles at a variant nucleotide position.

We applied quantitative minisequencing with tritium-labeled nucleotides ^3^H[dCTP] and ^3^H[dGTP] to determine the allele frequency of the major AGU mutation in a pooled DNA sample, consisting an equal amount of normal DNA from 1,350 individuals from the Finnish population. The method was highly sensitive, allowing for quantification of a mutated nucleotide in less than 1% of a DNA sample. The amount of mutant AGU in the pooled sample showed that the carrier frequency of the major AGU mutation was 1:36 in Finland, in accordance with the carrier frequency of 1:30 observed in individual population samples (26).

### Diagnosis of mitochondrial diseases

In collaboration with PhD student Anu Suomalainen, we used quantitative minisequencing for the identification of heteroplasmic mutations in the maternally inherited mitochondrial DNA (mtDNA). We analyzed mtDNA carrying the tRNA^8344^lysine C > T point mutation in the MERRF (Myoclonous Epilepsy and Ragged-Red-Fiber) syndrome in leukocytes from a large family. The incorporation of ^3^H[dCTP] showed the presence of the mtDNA mutation in eight family members, while incorporation of ^3^H[dTTP] revealed a mixture of normal and mutant sequence (heteroplasmy) in the patients. The percent of mutations in mtDNA varied between 9 and 72%, and the amount of mutated mtDNA correlated with the severity or age of onset of the MERRF disease (28). Similarly, we used solid-phase minisequencing for quantification of the tRNA^3243^ leucine C > T point mutation of mtDNA in patients with the MELAS syndrome (Mitochondrial Encephalomyopathy, Lactic Acidosis and Stroke-like episodes). In this mitochondrial disorder, we also observed a correlation between the severity of the symptoms of MELAS and the proportion of mutant mtDNA, as well as large differences in the degree of heteroplasmy between tissues in MELAS patients (29).

In 1993, Anu Suomalainen established the solid-phase minisequencing method for routine clinical diagnosis of mitochondrial diseases at the Helsinki University Hospital. In addition to detection of the presence or absence of the major mutations in MELAS, MERRF, Leber hereditary optic neuroretinopathy, and neuropathy ataxia and retinitis pigmentosa, the precise degree of heteroplasmy was determined in the patients and in asymptomatic disease carriers. Individuals with a low amount of mtDNA mutations have a small risk of disease, but female carriers may have a larger number of mutations in their ovaries due to a bottleneck during ovary development, which could lead to a selective increase of mutations in mtDNA in the off-spring. In a particularly interesting case, a non-symptomatic woman had undetectable mutations in her mtDNA, while her first child carried almost 100% of mutant mtDNA, while placental biopsy showed that her next child had 0% of the mutant mtDNA. Thus, the mitochondrial mutation in her first child occurred due to one mutant ovarian cell in the mother. In such cases, accurate and sensitive detection of mutations of mtDNA is crucial for diagnosis. The solid-phase minisequencing methods were used for routine diagnosis of mitochondrial diseases at the Helsinki University Hospital from 1993 until 2014, when next generation sequencing (NGS) of the complete mtDNA was implemented in the diagnosis of mitochondrial diseases.

## PCR-based forensic analysis

In 1984, Alec Jeffreys discovered that the human genome contains a large number of tandemly repeated ‘minisatellite’ regions (variable numbers of tandem repeats (VNTRs)), which are highly polymorphic between individuals. This finding paved the way for forensic DNA-profiling of individuals in criminal cases and for paternity testing (30). In 1989, the forensic laboratory at the National Public Health Institute undertook one of the earliest criminal case studies with PCR to analyze VNTRs markers close to the Apolipoprotein B gene. The study included two forensic cases with one murder victim in each case, and blood and stain samples from both suspects. Size analysis of the amplified PCR fragments showed the origin of the blood stains from one of the suspects in each case. This study was one of the first ‘real life’ forensic cases to be successfully solved using PCR (31).

**Figure 2:**
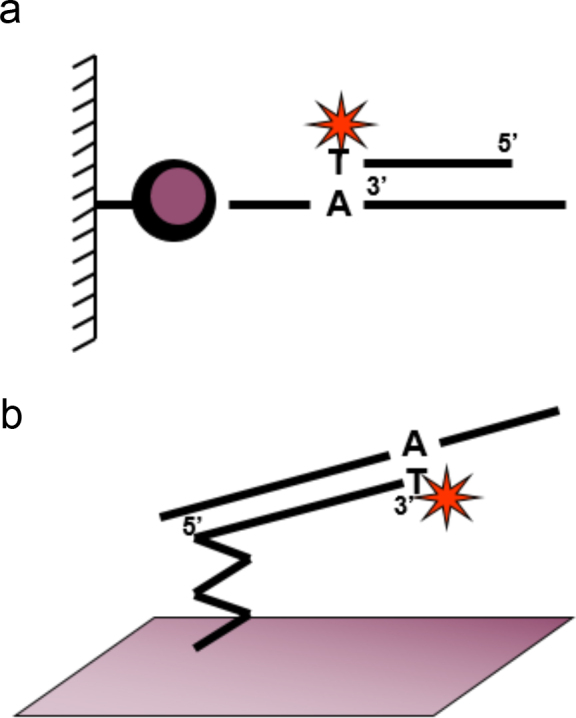
Illustration of two minisequencing assay formats: (a) Solid-phase minisequencing of a biotinylated PCR product immobilized in a microtiter well, followed by hybridization of a detection primer to the template and extension of the primer with a labeled complementary nucleotide. (b) Microarray minisequencing with a DNA template amplified in solution by PCR, followed by hybridization of the template to a detection primer immobilized on a microarray surface and extension of the primer with a labeled complementary nucleotide.

I was interested in developing a method for forensic identification of individuals based on analysis of SNPs using the solid-phase minisequencing method. PhD student Antti Sajantila and I selected a panel of 12 SNPs located in genes on different chromosomes with allele frequencies close to 0.5 for maximal information content. This study demonstrated the power of multiplex, robust SNP genotyping, and its quantitative applications, one of my favorite publications (32). We used a microtiter plate format for our method since it was suitable for automation, and the result of the assay was obtained as numerical values (Figure 2a). The probability of a random match in the forensic cases for the 12-marker panel used here and the power of exclusion in paternity testing were consistent with that of three VNTR markers combined with 10 protein markers that were used at our institute early in 1990. Most of the analyzed SNPs were located in the coding region of a gene. This was because in 1990, when the study was conducted, most known human DNA sequences were found in coding exons.

It later turned out that VNTR markers do not perform optimally in forensic PCR applications, mainly due to their long repeat sizes and frequent degradation of DNA in forensic samples (33). Consequently, forensic DNA-profiling using short tetra- or trimeric tandem repeat markers (STRs; microsatellites) with multiplex PCR became the method of choice for forensic PCR analysis and identification of individuals (34). Powerful multiplex panels of STRs based on fluorescence detection, capillary electrophoresis, and software for data interpretation have been developed since the 1990s, when STR markers were first described (35).

Today, STR marker panels constitute the core of the international forensic databases that have been instrumental for resolution of criminal cases all over the world. Hence, STR markers have remained as the primary markers for forensic genetics until today (2024). It is obvious that SNP markers will not replace identity testing by STR markers in forensic genetics (except for mtDNA). However, it will be interesting to see if or when NGS will become the method of choice in routine forensic analyses.

## Microarray technology for SNP analysis

Microarray technology for multiplex detection of gene expression levels was developed in the beginning of the 1990s, initially for model organisms (36) and later for human gene expression (37). Microarray-based analysis of gene expression was performed on a large scale in the late 1990s for analysis of thousands of genes in a single sample on a microscope slide (38), but SNP analysis in a microarray format was still not well established at this time.

## Solid-phase minisequencing in a microarray format

PhD student Tomi Pastinen developed a microarray system for multiplex SNP genotyping of disease-causing mutations by solid-phase minisequencing (Figure 2b) (39). We used the mutations of the Finnish recessive disorders as a model in our first study, in which we analyzed nine disease-causing mutations. Genomic fragments spanning the disease-containing mutations in each sample were amplified by multiplex PCR, and the PCR products were hybridized to the minisequencing primers in sub-arrays on a microscope slide. The primers were extended with ^33^P[ddNTPs] using a DNA polymerase. After washing, the microscope slides were scanned for signal detection by a phosphoimager. The low energy radiolabeled ^33^P[ddNTPs] allowed measurement of the genotype data at high sensitivity. In most cases, the difference between heterozygous and homozygous signals was two orders of magnitude. To comparison of the the minisequencing method with hybridization with immobilized allele-specific oligonucleotide probes in the same microarray format, rendered corresponding signal differences of three orders of magnitude (39). We concluded that this first installment of the microarray minisequencing system was promising for future developments.

### Allele-specific primer extension on microarrays

Tomi Pastinen developed an allele-specific primer extension method that simplified assay procedures and made it possible to use a single, fluorescent-labeled dNTP rather than four radioactively labeled ddNTPs (40). Compared to our original minisequencing method, Pastinen’s assay was faster and required only a single detection step for each SNP or mutation. It was suitable for a study with a moderate number of mutations in a large sample set.

**Figure 3:**
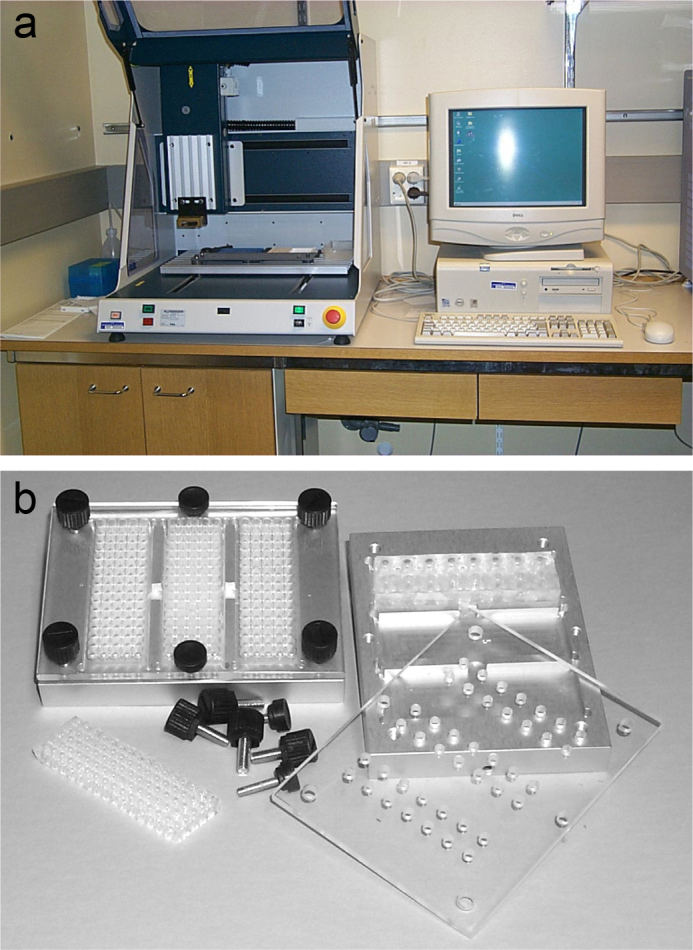
In-house manufactured modified robot for printing microarrays and silicon rubber reaction chamber for minisequencing reactions: (a) To automate printing of the minisequencing primers on a microscope, a low-cost industrial robot for etching a gluing (Isel Automation, Eiterfeld) was modified with an in-house manufactured tweezer-like tip for printing oligonucleotide primers in circular arrays on microscope slides in a format compatible with multichannel pipets. The robot had a capacity to print minisequencing detection primers for 300 samples per microscope slide. (b) Reusable miniaturized silicon rubber reaction chambers were prepared in-house molded on an inverted 384-well microtiter plate with V-shaped wells as a mold. Liquid silicon rubber (Elastosil RT 601 A/B, Wacker-Chemie GmbH) was poured into the mold, leaving about 1–2 mm of the tip of the wells uncovered. After allowing the rubber to harden over night, the grids containing 384 cone-shaped reaction chambers were cut to match the size of microscope slides. A rubber grid was placed over the primer arrays to form 80 separate reaction chambers. The reaction chambers had a glass surface with the primer array as bottom and the molded cone-shaped silicon rubber as wells, with open tops for pipet tips to fit into the chambers. Prior to adding the reaction mixtures, the rubber grid is firmly pressed against the glass surface in a custom-made aluminum rack with a Plexiglas cover containing drill holes for the pipet tips, through which the reaction mixtures are added. An aluminum rack, which can be heated, holds three microarrays with 80 reaction chambers.

**Figure 4:**
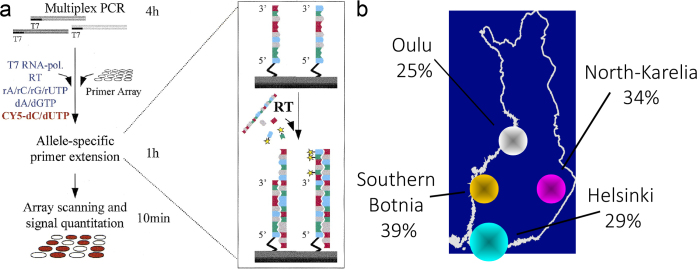
Principle of allele-specific primer extension and genotyping results by allele-specific primer extension: (a) A pair of allele-specific primers with different 3’-ends that are complementary to each mutant or variable SNP allele is immobilized on a microscope slide as small circular arrays (125–150 uM in diameter). The DNA templates are amplified by multiplex PCR. During PC, T7 RNA polymerase promoter is inserted into the 5’-end of the DNA fragments. For genotyping, the PCR products are added to the primer arrays together with ribonucleotides (rNTPs) and a T7 RNA polymerase to generate multiple RNA targets by reverse transcription of each PCR product. Simultaneously with reverse transcription, fluorescent CY5-labeled dNTPs are incorporated into the target molecules in allele-specific primer extension reactions. The fluorescent signals on the microscope slides are quantified using a confocal fluorescence scanner, and the data are interpreted with a custom-designed software. (b) Carrier frequencies of 31 mutations of the ‘Finnish disease heritage’ determined in 2,100 population samples from four geographical regions of Finland.

We designed a ‘Finnish Chip’ to determine the carrier frequencies of 31 mutations from the Finnish disease heritage and applied it for analysis of 2,100 population samples from four geographical regions of Finland. For this study, circular ‘array-of-arrays’ were printed on 80 microscope slides. To automate printing of the primers, we used a modified low-cost industrial robot, shown in Figure 3a. For the primer extension reactions, an in-house silicon rubber grid was placed on the microscope slides to form reaction chambers on top the ‘array-of-arrays’, as illustrated in Figure 3b. The steps of the assay are described in Figure 4a. We analyzed all samples in duplicate, which yielded 128,000 genotypes. The over-all success rate for the genotyping was 99.2% (40). The average carrier frequencies of the combined 31 mutations varied between the four regions in Finland, as shown in Figure 4b. An interesting result from this study is that one third of the genotyped individuals were carriers of at least one of the mutations of the Finnish disease heritage, which demonstrated the feasibility of multiplex population screening program for these mutations in Finland.

## Molecular medicine in Uppsala

In 1998, I transitioned from my job at the National Public Health Institute in Helsinki to Uppsala University, where my colleague Ulf Landegren had convinced the Medical Faculty to recruit an associate professor (universitetslektor) in Molecular Medicine to introduce genetics and genomics into clinical research at Uppsala University and Uppsala University Hospital. I was appointed to this position and joined the Department of Medical Sciences at Uppsala University, where my new lab was located centrally at the Research Department at the Hospital in Uppsala. The first lab members were two postdoctoral scientists, Maria Lagerström and Gisela Barbany, and a PhD student Charlotta Olsson, who relocated from Ulf Landegren’s laboratory to my lab at the hospital. They played a crucial role in helping me get started with my research in Uppsala, including setting up the laboratory and establishing the necessary infrastructure for our work. My first three PhD students joined the group during 1998–2000. Their research focused on developing a large-scale, robust SNP genotyping system using fluorescent detection in a microarray format and to apply it to clinical samples. In 2001, I was appointed to Professor in Molecular Medicine at Uppsala University.

## Wallenberg Consortium North

During the mid-1990s, there had been a strong demand from the scientific community for a national research infrastructure for Functional Genomics in Sweden. In 2000, the Knut and Alice Wallenberg foundation (KAW) decided to fund two major consortia for Functional Genomics: one for three universities in Southern Sweden (Swegen) and another for four universities located in Stockholm, Uppsala, and Umeå, collectively forming the Wallenberg Consortium North (WCN). The KAW funding of these two consortia was crucial for the largest scientific program in biomedicine ever undertaken in Sweden. It was a fortunate coincidence for me that I arrived in Sweden around the same time as WCN announced their call for research infrastructures and large-scale genomics projects.

The WCN infrastructure consisted of five technology platforms (SNP/DNA analysis, gene expression, proteomics, gene function *in vivo*, and bioinformatics). I was recruited by WCN to serve as coordinator for the SNP/DNA analysis platform. In the beginning of 2000, there was a great interest in developing and applying methods for SNP genotyping (21). The hope was that the analysis of SNPs would allow identification of genes that underlie complex human diseases. The funding from WCN in 2001 made it possible for the SNP/DNA technology platform to purchase state-of-the-art equipment for SNP genotyping to all four partner universities. The WCN SNP/DNA analysis platform established four nodes with expertise in different genotyping technologies, with Minisequencing at Uppsala University (UU), Pyrosequencing at KTH Royal Institute of Technology (KTH), and three hybridization-based assays MassArray (Sequenom) and dynamic allele-specific hybridization at Karolinska Institutet (KI), and TaqMan (Applied Biosystems) at Umeå University (UmU). The mission of our platform was to offer internationally cutting-edge SNP technologies as a service to the scientific community in Sweden (and other countries). Having recently moved to Sweden, the WCN infrastructure played a crucial role in integrating our research group into the Swedish genomics community and connecting us with a national network of scientists in the genomics field. The support by WCN was also important for our work, providing access to state-of-the-art equipment for SNP genotyping, which significantly advanced our research capabilities.

## The SNP technology platform in Uppsala 2001–2006

The first service project to be completed at our SNP Technology Platform was an evolutionary study on flycatcher speciation (41, 42). This project was carried out in collaboration with the Evolutionary Biology Centre at UU, using our in-house developed Tag-array minisequencing system (described below).

In the beginning of 2000, WCN granted 10 million SEK to Professor Hans Lithell and his coworker Lisa Byberg for a large study of genetic determinants of hypertension, insulin resistance, and diabetes within the Uppsala Longitudinal Study of Adult Men (ULSAM) project. The ULSAM study included all men aged 50 during 1969–1974 in Uppsala County and was led by Hans Lithell until 2005 (43). The genotype–phenotype studies within ULSAM are still in progress (2024) and have so far resulted in 200 publications with support from the SNP Technology Platform.

Other prominent large-scale human genotyping projects performed at the SNP technology platform in Uppsala were ‘Genome EU Twin’ (44), ‘Genetic epidemiology of breast cancer’ (45), ‘Genetic epidemiology of birth weight’ (46), and ‘Genetic factors in acute coronary syndrome project’ (FRISC II study) (47). Typically, these projects comprised relatively large sample sets, from 1,000 to 2,000 DNA samples from patients and controls, but only a small number of SNP markers.

Single SNPs were genotyped with a homogeneous fluorescence polarization assay (LJL Analyst AD) using single-base primer extension, established at the SNP Technology Platform by funding from WCN (48). For a small numbers of SNPs, we used our in-house developed solid-phase minisequencing assay in microtiter wells or the commercial 12-plex SNPstream system launched in 2003 (by Orchid Biosciences/Beckman Coulter) (49). The small number of SNPs in the genetic studies above is because at this time only a very limited number of verified SNPs were available in SNP databases.

To secure a high quality of the SNP genotyping process, the SNP Platform had established robust quality standards during 2003–2004, and in 2005, the platform was accredited by SWEDAC (the national accreditation body for Sweden). At the end of the WCN program 2006, the SNP Platform had a staff of 10 persons, including research engineers, laboratory technicians, and bioinformatics experts. Funding of 10 persons was challenging, but fortunately, Prof Ulf Pettersson, a strong proponent of genomics in Sweden, was able to provide interim funding from UU for the SNP Technology Platform.

## Multiplex fluorescent SNP genotyping on microarrays

My research group developed a microarray-based minisequencing method with fluorescence-labeled dideoxynucleotides (ddNTPs) for large-scale SNP analyses in-house. This work had begun already in Helsinki with PhD student Katarina Lindroos, who was involved in development of a microarray method for fluorescent genotyping of Y-chromosomal SNPs (50). She joined my group in Uppsala in 2000 and made important contributions to chemical immobilization of oligonucleotides on glass microscope slides (51). Below, we describe two examples of multiplex four color fluorescent SNP genotyping on microarrays published in the beginning of the 2000s.

### Minisequencing with immobilized SNP-specific primers

We used a microarray format on microscope slides for SNP genotyping with fluorescence detection using four dideoxy nucleotides (ddNTPs) labeled with Tamra (tetramethyl-6-carboxyrhodamine) in four separate reaction mixtures (NEN Life Science Products). Alternatively, we used four-color fluorescence labeling in a single reaction mixture of the four fluorescent nucleotides, Texas Red-ddATP, Tamra-ddCTP, R110-ddGTP, and Cy5-ddUTP (Perkin Elmer Life Sciences). We used the silicon rubber grid that had been developed by Tomi Pastinen (40), to create individual reaction chambers for each sample in an ‘array-of-arrays’ pattern on a microscope slide. The microscope slide held 14 PCR-amplified samples labeled with the same fluorescent dye in four lanes or 80 samples labeled with four different fluorescent labels in one lane.

Using four-color detection with immobilized SNP-specific primers, PhD student Ulrika Liljedahl established a method for quantitative determination of genomic DNA in mixed samples. The method was carried out by multiplex PCR, subsequent minisequencing with primers immobilized on a microscope slide, followed by the detection with four-color fluorescent ddNTPs (52). In a mixed sample, the sensitivity for detecting a minority allele was below 5% of that in the corresponding homozygous sample.

### SNP-genotyping by minisequencing in solution

**Figure 5:**
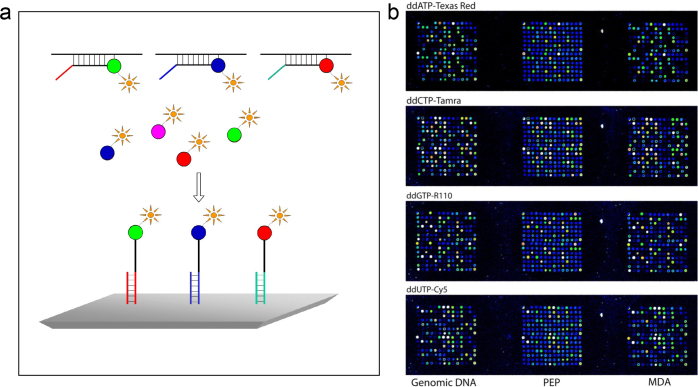
Principle of four color tag-array minisequencing of single nucleotide polymorphisms: (a) Each minisequencing primer contains a unique 5’-tag sequence for capture of the extended primers by an immobilized 3’-complementary (c-tag) oligonucleotide in an ‘array-of-array’ configuration on a microscope slide. C-tag oligonucleotides have been immobilized on CodeLink Activated microarray slides (Motorola) by mediation of a 3’-NH2 group using a ProSys 5,510 spotter (Cartesian). The SNPs to be genotyped are amplified by multiplex PCR, after which cyclic minisequencing reactions in solution are performed with four fluorescently labeled ddNTPs in which the extended detection primers anneal immediately adjacent to each polymorphic SNP position. After genotyping, the fluorescent signals are measured using a ScanArray 5,000 instrument (Perkin Elmer Life Sciences), and the genotypes are assigned using the QuantArray^®^ analysis software of the instrument. (b) Images obtained by scanning a microscope slide at four wavelengths from one individual genotyped by WGA for a panel of 45 SNPs using tag-array minisequencing. Result from primers in both DNA polarities at duplicate positions is shown. The images from genomic DNA (WGA) and primer extension preamplification (PEP) and MDA products are shown in three vertical rows. The fluorescent labels used for the four dideoxy-dNTPs are indicated above the horizontal rows of the subarrays. The obtained signals are reproduced with an artificial rainbow scale with blue as low and white as saturated signal.

For multiplex genotyping, the SNPs are subjected to multiplex PCR, after which cyclic minisequencing reactions in solution are performed with four fluorescently labeled ddNTPs, and the extended primers are captured on a microscope slide in an ‘array-of-arrays’ configuration (Figure 5a). A major advantage of minisequencing in solution is that the immobilized-capturing oligonucleotides are generic and directly applicable for genotyping any SNPs and mutations without customized printing of specific primer oligonucleotides. Another advantage is that the kinetics of the minisequencing reactions are faster in solution than on a solid support. The ‘array-of-arrays’ format with four-color fluorescence detection allows genotyping of up to 80 individual samples per microarray slide with up to 100 spots per subarray to generate 8,000 genotypes per slide. Multiplex ‘tag-array’ minisequencing became the in-house method of choice in many research projects in our laboratory. It was also used by the SNP technology platform in several of the early service projects, before commercial genotyping equipment became available.

PhD student Lovisa Lovmar applied four-color multiplex tag-array minisequencing for quantitative evaluation of whole genome amplification (WGA) for producing a large amount of genomic DNA. She compared genomic DNA with two WGA methods, multiple displacement amplification (MDA), and primer extension preamplification (PEP), by analyzing 45 SNPs located in different genomic regions in duplicate and in both polarities (Figure 5b). The SNPs were analyzed in DNA samples from 15 three-generation family members from the CEPH collection (Jean Dausset-Centre dEtude du Polymorphisme Humain Nacional in Paris). No Mendelian inheritance conflicts were detected in the three-generation CEPH family samples, indicating a high accuracy of the method. In total 45,000 fluorescent signals were generated in this experiment. The overall success rate for the genomic DNA samples was 97.0%, i.e. 655 genotypes out of a maximum of 675 were called, and the result from MDA was concordant with the result from genomic DNA in 99.7% cases. The corresponding number for PEP was 88.7%. We concluded that WGA, and MDA in particular, is a highly promising tool for obtaining unlimited amounts of genomic DNA for large-scale SNP genotyping studies (53).

## Association of type-1-interferon genes with systemic lupus erythematosus

Systemic lupus erythematosus (SLE) is a chronic autoimmune disease in which the immune system mistakenly attacks the body’s own tissues and organs. It can affect multiple systems in the human body, leading to widespread inflammation and tissue damage. Type I interferon (IFN) play a crucial role in the pathogenesis of SLE by promoting immune activation and sustaining chronic inflammation. Thus, we were inspired by the important role of the type-1-IFN system in SLE (54) to perform a candidate gene association study with as many verified SNPs as possible. We identified 44 SNPs in 11 candidate genes from the type-1-IFN system and genotyped these SNPs in 679 Swedish, Finnish, and Icelandic patients with SLE and 1,236 unaffected controls. Today, this number of SNPs appears low, but at this time, most genetic association studies were performed using a single SNP or only few SNP markers. In this large project, PhD student Snaevar Sigurdsson performed multiplex genotyping using the tag-array minisequencing method described above (55).

Because our DNA samples comprised both pedigree samples and singletons, we performed joint statistical linkage and association analysis of each individual SNPs using the Pseudomarker method (56). Pseudomarker analysis unveiled a strong association signal for two linked SNPs in the coding region of the Tyrosine kinase 2 (*TYK2)* gene and several linked SNPs in the promoter region and the first intron of the Interferon regulatory factor 5 (*IRF5)* gene. The *P*-value was 4.2 × 10^-8^ for the top *TYK2* SNP (rs2304256), and the *P*-value was 5.2 × 10^-8^ for the top *IRF5* SNP (rs 20004640) (55). It is remarkable that in this study, published in 2005, we identified two genes with strong association signals for SLE among only 11 analyzed candidate genes with a connection to the type-1-IFN system. It is also notable that we obtained very strong signals of association (*P*-value ~10^8^) for the top SNPs in the patients with SLE.

Shortly after this result, we also identified an equally strong association signal for SLE in the Signal transducer and activator of transcription 4 *(STAT4)* gene (57). We found that 10 out of 53 genotyped SNPs in *STAT 4* were associated with SLE with a *P*-value of 7.1 × 10^-8^ for two top linked SNPs (rs101811656 and rs7582694). By allele-specific gene expression (ASE) analysis, we found that the risk allele of *STAT4* was overexpressed in human primary mesenchymal cells (but not in B-cells) and in cells carrying the risk allele of *STAT4,* compared with cells without a risk allele. Later, the *IRF5, STAT4*, and *TYK2* genes have been replicated all over the world in genome-wide association studies (GWASs) of SLE and several other diseases of the immune system.

In collaboration with Prof Timothy Behrens at Genentech in the USA, we contributed to an early GWAS of SLE, published in 2008, by genotyping two SNP loci in around 800 samples from Swedish SLE patients and 850 Swedish controls. Inclusion of the data from the Swedish samples secured formal genome-wide statistical significance (*P*-value < 10^-8^) for the previously unknown *FAM167* (C8orf13)-*BLK* and *ITGAM-ITGAX* loci as risk factors for SLE (58). Genotyping of the two SNP loci of interest was performed by post doctor Sophie Garnier in my lab, using minisequencing in a homogeneous fluorescence polarization assay (described above) (48).

### National network for genetic studies of SLE

Our SLE studies led to a productive long-term collaboration in genetics between the Molecular Medicine group and Professors Lars Rönnblom and Gunnar Alm, and the Rheumatology group in Uppsala. In 2003, a national SLE network for genetic studies had been established between the Swedish university hospitals, which gave us access to a large number of well characterized patient samples from Swedish patients with SLE. We contributed to several international and local Swedish association studies that identified SLE genes, exemplified by the identification of the *TNIP1, PRDM1, JAZF1, UHRF1BP1*, and *IL10* genes (59) and the *IKBKE* and *IL8* genes (60) as risk loci for SLE. In total, the Swedish SLE network has so far contributed to the identification of >100 genetic associations in SLE.

## Functional analysis of SLE genes

### Chromatin immunoprecipitation

**Figure 6:**
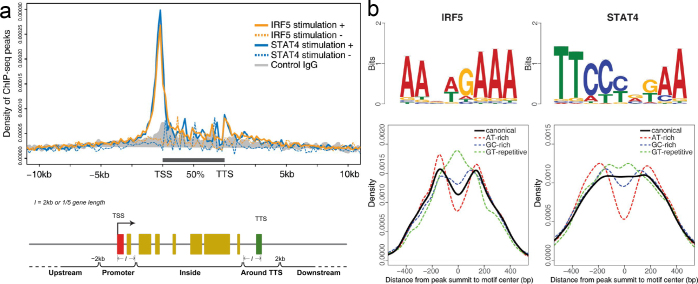
Chromatin immunoprecipitation sequencing (ChIP-seq) to detect target genes for the SLE-associated transcription factors *IRF5* and *STAT4*: (a) Locations and annotations of ChIP-seq peaks for *IRF5* and *STAT4*. The positions relative to the annotated genes before filtering are shown for IRF5 (orange) and STAT4 (blue). (b) Binding motif analysis in the target regions for transcription complexes containing *IRF5* and *STAT4*. Logos for the canonical binding motifs for *IRF5* and *STAT4* in their target regions are shown. Distribution of the distances between the summits of ChIP-seq peaks and the center of the canonical IRF5 and STAT4 binding motifs (solid lines), and the three most common motifs for other proteins identified in the ChIP-seq peaks (dashed lines).

The genes *IRF5* and *STAT4* encode transcription factors that cause a major risk for SLE. PhD student Chuan Wang used chromatin immunoprecipitation (Chip-seq) to determine the target genes for IRF5 and STAT4 in stimulated human peripheral blood mononuclear cells (PBMCs). Chip-seq analysis revealed more than 7,000 target genes for IRF5 and STAT4 that are enriched in functional pathways in the type 1 IFN-system and have key roles in the inflammatory response in stimulated PBMCs. IRF5 and STAT4 are part of the same transcriptional complexes, and the transcription factors HMG-I/Ym, SP1, and PAX4 regulate a large number of genes in stimulating PBMCs, which may explain the strong association of IRF5 and STAT4 with SLE (Figure 6) (61). Several of the target regions of IRF5 and STAT4 contain sequence variants that are associated with SLE and may influence the transcriptional complexes containing *IRF5* and *STAT4*. Annotation of target genes by ChIP-seq, which is independent of linkage equilibrium, could provide alternative functional hypotheses for the association signals in putative risk loci and plausible candidate genes located in the vicinity. *IRF5* gene variants that confer risk of SLE seem to be correlated with increased expression of type 1 IFN, while the functional variants of *STAT4* are predominantly associated with enhanced expression of genes induced by type-1-IFN.

### Random forest classification

Jonas Carlsson Almlöf, a senior bioinformatician who joined my lab in 2013, introduced a machine learning method based on random forests to design an SNP genotype classifier for the identification of previously unknown risk genes for SLE and for prediction of an individual’s risk for SLE (62). About 135,000 SNPs located in 12,500 genes relevant for SLE included on the ‘Immunochip’ (Illumina Inc.) were genotyped in 1,160 patients with SLE and 2,710 controls. Among the 40 genes with top risk scores for SLE defined by the random forest classifier, we identified 15 potential risk genes for SLE. Of them, 3 genes, *ZNF804A, CDK1*, and *MANF,* were strong candidate genes for SLE based on their involvement in pathways affected in SLE. Functional validation by ASE analysis of 50 blood donors identified about 700 genes with ASE in B- and/or T-cells. Thirty of the 40 top genes were expressed in B- and/or T-cells, and of them, six genes were regulated by *cis*-acting regulatory SNPs (*cis*-rSNPs). The *cis*-rSNPs for the SLE-associated genes *IKZF1, NCF2, IL2A, TNIP1*, and *PHRF1* in B-cells, and *ANK3* and *PHRF1* in T-cells suggest a functional role for them in SLE.

### DNA methylation mapping in SLE

To explore the role of epigenetics for the risk of acquiring SLE, we analyzed DNA methylation profiles in a large collection of patients with SLE. In a collaboration between the Rönnblom group and my group, post doctor Johanna Sandling analyzed 548 patients with SLE and 587 control persons using the Methylation BeadChip (Illumina Inc.), which features ~450,000 CpG sites across the genome. SNP genotype data from the ImmunoChip (Illumina Inc.) was used for methylation quantitative trait loci (*cis*-meQTL)-analysis (63). Over 7,000 differentially methylated CpG sites were identified with the largest effect being a decrease in the methylation levels of type 1 IFN-regulated genes. The meQTLs for CpG sites in SLE were enriched for genetic associations with risk loci for SLE (*PTPR, MHC-class III, UHRF1BP1, IRF5, IRF7, IKZF3*, and *UBE2L3*). The results from this study, published in 2018, suggest that alterations of DNA methylation levels in regulatory regions of target genes significantly affect the SLE-phenotype.

## Primary Sjögren’s syndrome

### Genetic association studies

Primary Sjögren’s syndrome (pSS) shares several features with SLE. To identify shared and unique features of pSS and SLE, Professor Gunnel Nordmark and her coworkers performed genetic studies of pSS. The *IRF5* and *STAT4* genes that are strong risk factors of the type I IFN system in SLE are also important as risk factors for pSS. In accordance with the important role of B-cells in pSS, we found that the *EBF1* gene, the *FAM167A-BLK* locus, and the *TNFSF4* gene are involved in B-cell differentiation and activation of the immune system and thereby contribute to the pathogenesis of pSS (64).

### Epigenetic regulation of gene expression

Gunnel Nordmark and PhD student Juliana Imgenberg-Kreuz performed genome-wide DNA methylation analysis in pSS using the 450k Methylation Bead Chip (Illumina Inc.). DNA methylation was analyzed in three tissues: whole blood, CD19+ B cells, and in minor salivary gland biopsies from patients with pSS. In parallel, gene expression was analyzed in CD19+ B cells by RNA sequencing. We found that IFN-regulated genes were hypomethylated in whole blood and CD10+ B cells in the *MX1, IFI44L*, and *PARP* genes, while the IFN-induced *OAS2* gene was hypomethylated in salivary gland biopsies (65). These observations revealed previously undescribed associations between hypomethylation of IFN-regulated genes and increased gene expression in B-cells at known risk loci for patients with pSS.

We also performed a comparative analysis of patients with SLE and pSS with the 450K Methylation Bead Chip, using a random forest machine learning classifier trained to predict disease status based on DNA methylation data. Differential methylation analysis between SLE and pSS showed decreased methylation in pSS compared to SLE. Although most DNA methylation patterns are shared between SLE and pSS, there are important quantitative differences between the two diseases. Our study, which was published in 2019, provides evidence for genes and pathways driving both common and disease-specific pathogenic mechanisms in SLE and pSS (66).

### Multimodal single-cell sequencing of B cells

B-cells are essential for the pathogenesis of pSS, and we performed single-cell sequencing for detailed knowledge of the B-cell composition, gene expression, and B-cell receptor usage in patients with pSS. Gunnel Nordmark led the study, and post doctor Gustav Arvidsson performed the data analyses together with senior bioinformatician Paulo Czarnewski. More than 230,000 B cells were isolated from peripheral blood of 24 pSS patients and four healthy controls, after which the cells were processed for single-cell RNA sequencing (scRNA-seq) and single-cell variable, diversity, and joining (VDJ) gene sequencing (scVDJ-seq).

**Figure 7:**
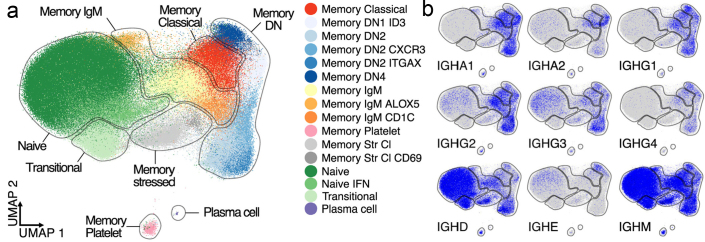
Multimodal single-cell sequencing of B cells in primary Sjögren’s syndrome (pSS): (a) Gene-expression-based UMAP (Uniform Manifold Approximation and Projection) representation of 232,102 B cells and 89 plasma cells. The colors indicate B cell types assigned by KNN (nearest neighbor) clustering. (b) Transcript expression levels for the immunoglobulin heavy chain (*IGH*) constant genes. Elevated transcript levels for *IGH* constant genes are indicated by a strong blue color.

Libraries for scRNA-seq and scVDJ-seq (8,000 cells per sample) were prepared and sequenced using protocols and equipment from 10X Genomics, aiming at 50,000 scRNA sequence reads and 5,000 VDJ-seq reads per cell. With the help of the scRNA-seq data, we assigned the individual B cells into five main subtypes and 16 cellular states with distinct circulating B cell composition (Figure 7a). The largest difference between patients and controls was observed in the Sjögren syndrome antigen A / Sjögren syndrome antigen B -positive (SSA/SSB+) pSS patient group. This group displayed the highest proportion of naïve and memory B cells and the highest up-regulation of type 1 IFN-induced genes across all B cell subtypes. Memory B cells from SSA/SSB+ pSS patients had a higher proportion of cells with non-mutated VDJ transcripts than other pSS groups and controls (67) (Figure 7b). This study provides clues to differences between individual B cells that affect the phenotype and outcome of pSS. Compared to bulk B cells, single-cell analysis improves the resolution for stratification of patients with pSS.

This study was initiated in 2019 just before the outbreak of the COVID-19 pandemic, which delayed completion of the project until 2024. It resulted in what probably is my last major scientific publication.

## Genomes and epigenomes in acute lymphoblastic leukemia

We were lucky to have recruited Gudmar Lönnerholm to our research group after his long career as clinician and professor at the Department of Women’s and Children’s Health at the University Children’s Hospital in Uppsala. Pediatric oncologists in the Nordic countries were pioneers in biobanking in the early 1990s, when they formed the Nordic Society for Pediatric Hematology and Oncology (NOPHO) to enroll patients for clinical studies. Since pediatric ALL is a rare disease, the Nordic countries joined forces to have a sufficient number of samples for clinical studies of ALL. ALL is a heterogeneous disease in which patients are stratified into subtype groups based on their cellular immunophenotype and abnormal recurrent cytogenetic rearrangements creating fusion genes, in which part of two genes become joined. Starting in 2005, we analyzed samples from ALL patients from the Nordic countries in collaboration with Gudmar Lönnerhom and his coworkers. Pediatric ALL became a major research project in our group. Our aim was to apply emerging tools for large-scale genomics and epigenomics to improve and simplify stratification of patients with pediatric ALL into more specific treatment groups. Several PhD students and postdoctoral students in our group participated in the studies on ALL. Some of these studies are highlighted below.

### ASE and DNA methylation in ALL cells

**Figure 8:**
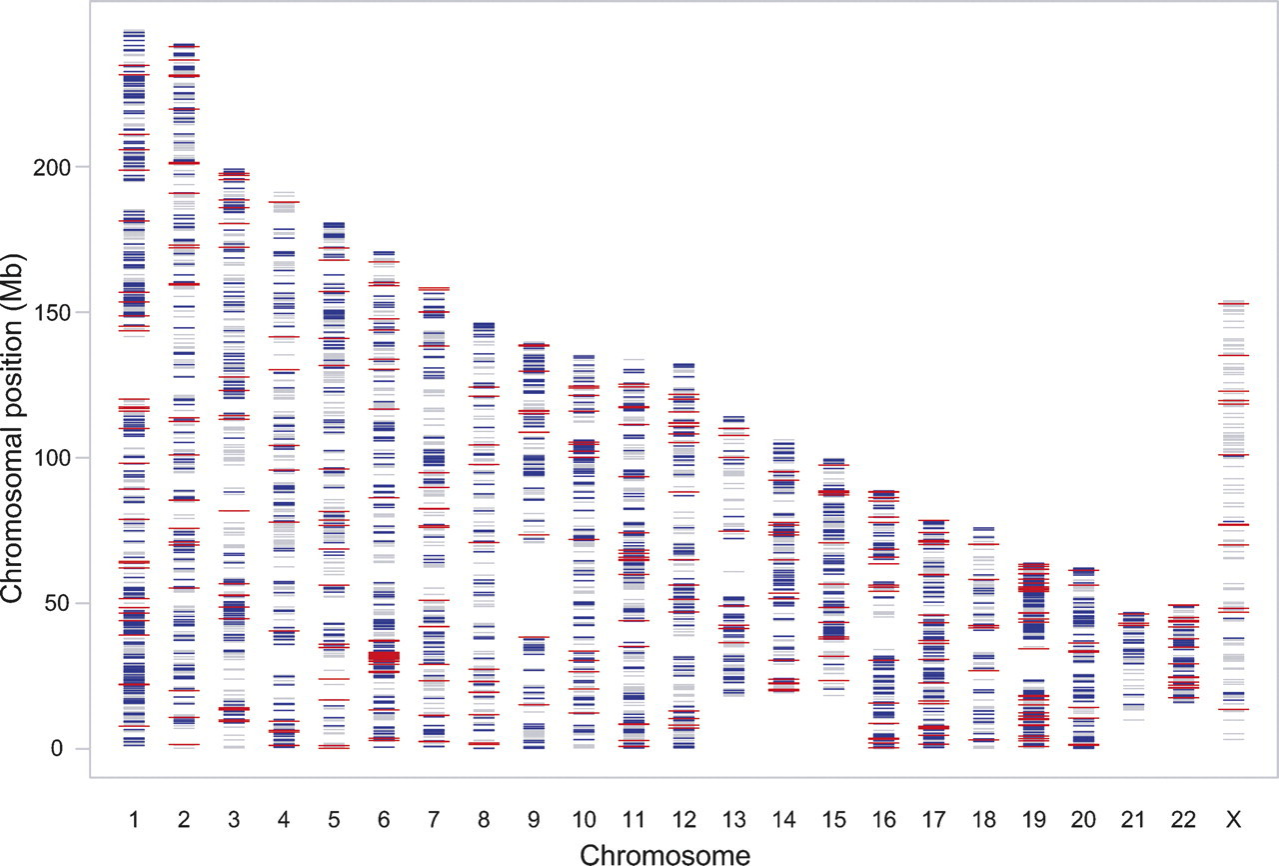
Genome-wide allele-specific gene expression in primary leukemic cells. Genome-wide distribution of 8,000 genes included on the Illumina NS-12 BeadChips (gray), 2,529 genes carrying heterozygous SNPs expressed in the ALL cell samples (blue), and 400 genes for which allele-specific gene expression was detected (red). The chromosome numbers are given on the *x*-axis and the chromosomal positions (Mb) on the *y*-axis.

PhD student Lili Milani and bioinformatician Anders Lundmark performed an innovative study, published in 2010, in which ASE in primary ALL cells was used to explore regulation of gene expression by *cis*-acting DNA methylation in gene-promoter regions (68). The study included bone marrow or peripheral blood samples from 197 pediatric ALL patients who were enrolled on the NOPHO ALL 1992 and ALL 2,000 treatment protocols. ASE levels were measured in the ALL cells by genotyping 14,000 SNPs in 8,000 genes in DNA and RNA (cDNA) using the Infinium assay and NS-12 BeadChips (Illumina Inc.). To be informative for ASE, an SNP has to be heterozygous in DNA and expressed at a detectable level in RNA. Of the SNPs on the BeadChip, ASE was robustly detected for 470 SNPs in 400 genes in at least eight samples. ASE turned out to be common in the ALL cells, and the genes with ASE were evenly distributed across the autosomal chromosomes (Figure 8). For most genes, the level of ASE varied between samples from 1.4-fold overexpression to monoallelic expression. An interesting observation supporting the existence of distinct gene regulatory mechanisms in different ALL cells was that the same allele was overexpressed in 45% of the genes (one-directionally), while for 55% of the genes, the overexpressed alleles varied randomly between samples (bi-directionally).

Lili Milani designed a customized DNA methylation analysis panel for 1,536 CpG sites using BeadChips from Illumina. The panel was used for quantitative analysis of CpG sites upstream or in the first intron of 386 genes in the same 197 ALL samples that were analyzed for the determination of ASE. We found that genes with bidirectional ASE had higher variability and higher levels of ASE than genes with one-directional ASE. In genes with bidirectional ASE, levels were correlated with the CpG site methylation levels. This observation is consistent with random methylation of the two alleles and shows that CpG site methylation is one of the factors that regulates gene expression in ALL. Surprisingly, using supervised learning by nearest shrunken centroid classification of variable CpG sites, we could differentiate between T-ALL and B cell precursor ALL (BCP-ALL) and could furthermore distinguish between the four major subtypes of BCP-ALL known at the time (69). We concluded that differential DNA methylation profiles are promising candidate markers for ALL subtype differentiation.

### Genome-wide signatures of differential DNA methylation

**Figure 9:**
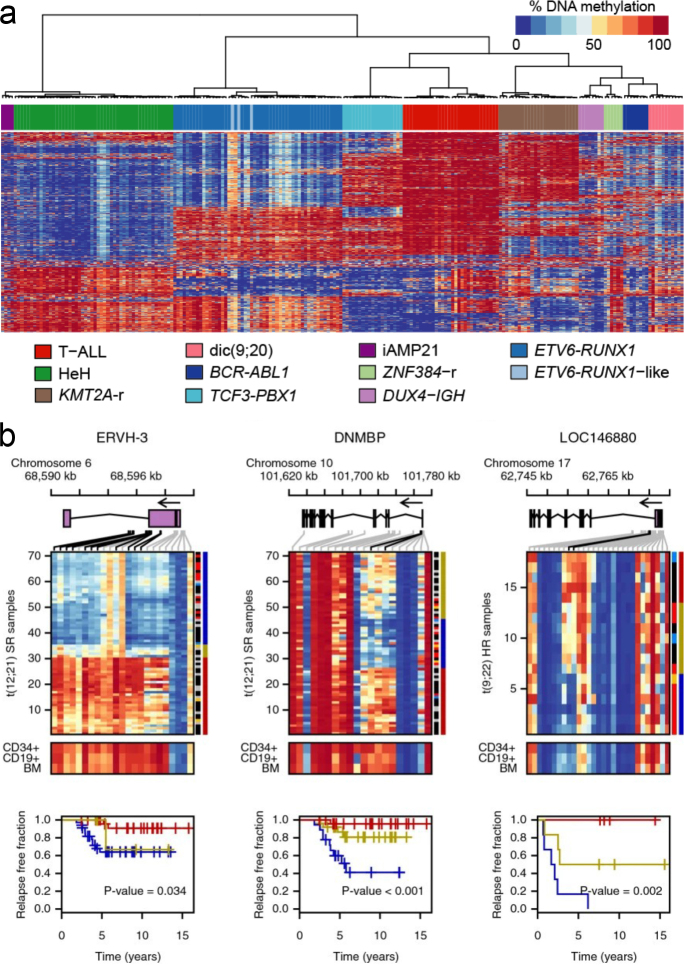
Heatmap of DNA methylation in ALL and prediction of relapse-free survival at differentially methylated CpG sites: (a) Hierarchical clustering of ALL and reference samples based on the methylation levels of ~436,000 CpG sites. The 1,000 most variable CpG sites are shown in the heat-map. (b) Methylation levels across the *ERVH-3* gene and *DNMBP* gene in patients with the t(12;21) *ETV6/RUNX1* translocation, and the precursor microRNA gene (LOC146880/ENSG00000215769) in patients harboring the t(9;22)*BCR/ABL1* translocation. Black lines above the heat-maps show differentially methylated CpG sites associated with relapse-free survival (*P* < 0.05). The patients (rows) are clustered based on the CpG sites associated with relapse-free survival. The outcome for individual patients is marked next to the heat-map with patients in remission in black, relapsed patients in red, late relapsed patients in yellow, patients with events other than disease relapse in blue, and patients censored before 5 years of follow-up time in gray. The average methylation levels in the non-leukemic controls are shown below the heat-map. At the bottom of each panel, Kaplan-Meier curves are color-coded by methylation groups, with blue indicating hypomethylation, yellow indicating intermediate methylation, and red indicating hypermethylation. The Kaplan-Meier curves demonstrate the difference in relapse-free survival of patients with different methylation profiles with the Gray’s test *P*-value for the difference shown in each panel.

PhD students Jessica Nordlund and Christofer Bäcklin embarked on the project to dissect the patterns of differential methylation in all subtypes of ALL on a genome-wide scale, using large-scale genomic analyses and advanced bioinformatics methods. This was one of the first genome-wide studies of DNA methylation. It encompassed ALL patients from the Nordic countries, with 663 patients with BCP-ALL including multiple samples from rare subtypes, and 101 patients with T-ALL. The samples were analyzed using the Infinium Methylation BeadChip assay (Illumina Inc.) by which ~450,000 CpG sites were quantified across the genome. Four DNA methylation signatures were revealed by the analysis. First, the methylomes of the ALL cells shared ~9,400 hypermethylated CpG sites, independently of cytogenetic subtype. Second, unique signatures of hyper- and hypomethylated CpG sites were identified in each cytogenetic ALL subtype (Figure 9a). Third, subtype-specific differential methylation pattern in regulatory gene regions was strongly correlated with gene expression. Fourth, a signature of ~6,600 CpG sites was found to be hypermethylated (increased in methylation) at ALL relapse. Analysis of relapse-free survival identified CpG sites with subtype-specific differential methylation that divided the patients into risk groups according to their methylation status (Figure 9b) (70). We concluded that DNA methylation plays an important role for gene regulation across ALL subtypes and may be informative of clinical outcome after treatment. Our results from this genome-wide DNA methylation study, gained an international interest, evidenced by frequent data request and citations (188 requests during 2013–2024).

### Refined ALL subtypes and novel fusion genes by DNA methylation

Jessica Nordlund and her coworkers went on to design a machine learning classifier for DNA methylation profiling for prediction of the eight canonical cytogenetic subtypes of pediatric ALL. After repeated cross validation, a final classifier comprising 246 CpG sites with high sensitivity and specificity was designed and applied to 210 ALL patients who lacked a clinically defined cytogenetic ALL karyotype (71). In about half the patients, a methylation profile was directly assigned to a known recurrent cytogenetic group. The DNA methylation-based subtype predictions were subsequently verified by methods such as karyotyping, copy number analysis, or RNA sequencing. In the remaining ALL patients for whom cytogenetic subtype and methylation classification did not agree, we discovered several previously unknown fusion genes involving key ALL genes such as *ETV6, RUNX1*, and *PAX5* genes. In 2024, we published an updated DNA methylation classifier for ALL, which now comprises 379 CpG sites and can accurately predict the subtype 17 of the known molecular subtypes of ALL (72).

PhD student Yanara Marincevic-Zuniga sequenced the transcriptomes of 134 patients with ALL to detect additional novel fusion genes. Seventy-four BCP-ALL patients with known recurrent subtypes, 42 BCP-ALL patients who lacked a defined cytogenetic subtype, and 18 patients with T-ALL were analyzed. Novel fusion genes were detected by a combination of genome-wide DNA methylation analysis, RNA-sequencing, and targeted sequencing (73). After filtering and experimental validation, 64 unique fusion events corresponding to 136 fusion genes, of which more than half had not previously been described in ALL, were identified in 80 patients. Twenty-one of the fusion genes were recurrent, and we identified *TTYH3-PDGFA, PDGFRA-SF1, VASH2-AFT3, CD69-HIST1H2BG, NCOR2-BCL7A*, and *P2RY8-CD99* as novel recurrent fusion genes in ALL.

### Sequencing of whole ALL genomes

Our sequencing of whole ALL genomes was initiated in 2010, just when NGS had made it possible, but no algorithms or automated pipelines for analyzing whole-genome sequencing (WGS) data from cancer cells were yet available. PhD student Mårten Lindqvist and Dr. Eva Berglund undertook the monumental task of manually developing methods for WGS data analysis and performing the bioinformatics analyses.

**Figure 10:**
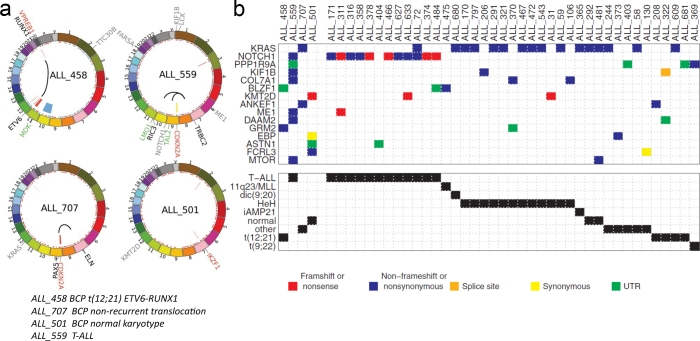
Four whole-genome sequenced ALL genomes and recurrent somatic mutations identified in a validation cohort: (a) Circos plots showing the genomic location of validated somatic single nucleotide variants (SNVs), insertion-deletions (indels), CNAs, copy neutral loss of heterozygosity (LOH), and translocations in four whole genome sequenced ALL patients. SNVs and indels are shown as red (original clone) or blue (subclone) dots in innermost circle of the chromosomes. Deletions are shown with red, duplications with blue, and LOH with yellow circle segments. Black arcs indicate translocations. Names for expressed genes with exonic indels or non-silent SNVs predicted to be damaging or putative drivers in the validation cohort (grey letters); genes involved in translocations (red letters); genes in CNA or LOH regions (black letters); differentially expressed genes that are located near breakpoints for translocations or putative regulatory SNVs (green letters). (b) Recurrent mutations in genes identified by whole-genome sequencing (WGS) in a validation cohort of ALL samples. Each column represents one patient, with the samples from WGS in the four leftmost columns. In the upper panel, each row represents one gene. Samples and genes with at least one mutation in an exon, splice site, or untranslated region (UTR) in the validation cohort are shown. Each colored box indicates a mutation. For patients with more than one variant in the same gene, the color is given in the order shown in the key below the panel. The genetic subtypes of the patients are given in the lower panel.

The genomes of four patients were sequenced to represent pediatric ALL. Three of the patients had BCP-ALL. Patient ALL_458 carried the t(12;21) *ETV6::RUNX1* translocation, patient ALL_707 had non-recurrent translocations, and patient ALL_501 had a normal karyotype. In the genome of one patient with T-cell ALL (T-ALL), patient ALL_559 with two translocations t(7;9) and t(7;11) was also sequenced. We sequenced the genomes of diagnostic and remission (normal) samples from these four patients to an average 31-fold coverage of each DNA base (Figure 10). By WGS, we found that each patient had a unique combination of known and previously undetected genomic aberrations, including SNVs, copy number alterations (CNAs), and fusion genes (74).

By RNA sequencing, we found that 10 out of the 23 genes with validated somatic exonic variants in the four patients sequenced by WGS were expressed as fusion genes. In addition to the canonical *ETV6-RUNX1* fusion gene in ALL_458, the expression of the rarely observed reciprocal fusion gene *RUNX1-ETV6* was detected. In ALL_559, the translocation t(7;9) and t(7;11) resulted in expression of two fusion genes with *TRBC2* as fusion partners in the T-cell receptor beta locus. According to karyotype data, ALL_707 had a t(7;9) translocation that results in a highly expressed *PAX-ELN* fusion gene. Based on these results, we concluded that the heterogeneity of the genetic aberrations in ALL makes WGS particularly well suited for analysis of somatic variations.

### Deep sequencing of somatic cancer mutations

We characterized the mutational patterns in ALL by deep-targeted sequencing using the HaloPlex target enrichment method (Agilent) by sequencing the exons of 872 cancer genes in 172 ALL patients of different diagnostic subtypes and of a subset of 19 patients who had relapsed. The high sequencing depth (638-fold) allowed detection of somatic mutations in diagnostic samples and allele fractions corresponding to somatic sub-clones in the diagnostic cells. By HaloPlex enrichment, we investigated if mutations identified at relapse were already present as minor sub-clones at the time point of diagnosis or if they were relapse-gained. In T-ALL, the mutations had occurred mainly in the original leukemic clone, while most of the mutations in BCP-ALL were subclonal. BCP-ALL patients with the recurrent translocations *ETV6::RUNX1, BCR::ABL1*, and *TCF3::PBX1* carried few driver mutations compared to other BCP-ALL patients. By computational prediction, we identified *ATRX* as a novel driver gene. Based on its protein sequence and structure, it was likely that *ATRX* affects the encoded protein. In T-ALL, the most frequent driver mutations were located in *NOTCH1,* and in BCP-ALL, they were in the RAS signaling pathway (*NRAS, KRAS, PTPN11*, and *FDLT3)* (75).

### Clonal evolution from diagnosis to relapse

We performed a comprehensive WGS study of the patterns of somatic mutations and clonal evolution of ALL cells. Under the leadership of post doctor Shumaila Sayyab, we sequenced 96 whole genomes from matched diagnostic, relapse, and remission (germline) DNA samples from 29 Nordic patients with ALL. Our sample set comprised 24 BCP-ALL samples, four T-ALL samples, and one sample with mixed phenotype leukemia. Somatic point mutation and large structural aberrations were identified by comparison with matched germline (remission) samples in samples from ALL patients.

**Figure 11:**
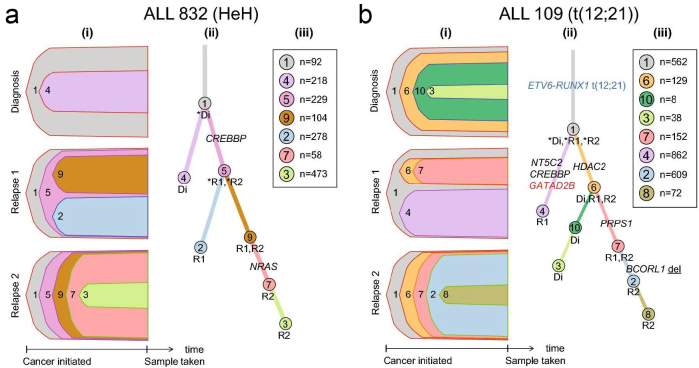
Clonal evolution in two examples of whole-genome sequenced patients with ALL: (a) Consensus model for clonal evolution in patient ALL_832 with high hyperdiploid (HeH) BCP-ALL. (b) Consensus model for clonal evolution in patient ALL_109 with the t(12;21) *ETV6-RUNX1* translocation. Both the figures sections (i) show clones and subclones at diagnosis, first, and second relapse, where clone 1 (in grey) is the founding clone. The size of the colored fields corresponds to the proportion of clones in a sample. Section (ii) shows clones and subclones at diagnosis (Di), first relapse (R1), and second relapse (R2). Each color-coded branch shows known and putative driver genes at each time point, with fusion genes in blue letters and regulatory non-coding genes in red letters. Section (iii) shows the total number of SNVs in each clone using the same color-code as in (i) and (ii). Driver mutations, somatic small insertion-deletions, putative regulatory non-coding mutations, copy number alterations, and structural variants are shown on the branches of the evolutionary trees.

We examined how the ALL cell populations evolved from diagnosis to relapse and identified three distinct patterns of clonal evolution in our ALL samples, denoted ‘persistent’, ‘rising’, and ‘founding’ clone trajectories. In the ‘persistent clone trajectory’, the main clone at diagnosis persists and had acquired additional mutations at relapse. Three of the patients were assigned to this trajectory. No fusion genes were detected in this trajectory (Figure 11a). In the ‘rising clone trajectory’, a subclone present at diagnosis expanded and acquired additional somatic mutations, after which it emerged as the major clone at relapse. In this trajectory, 9 out of 12 of the patients harbored large structural aberrations that resulted in expressed fusion genes (Figure 11b). Three patients had biallelic loss of one allele and carried a non-silent mutation on the other allele. In the ‘founding clone trajectory’, the allele fraction of non-synonymous mutations in driver genes increased from diagnosis to relapse. The clones at relapse were derived from an ancestral founding clone or clones that are ancestral relative to the clones present at diagnosis. Eleven patients were assigned to this trajectory (76). Our observations support the hypothesis that rising clones are resistant to chemotherapy, while the founding clones acquire additional mutations before emerging as major clones at relapse. In both models, relapse originates from clones that resisted therapy.

## The SNP&SEQ technology platform in Uppsala 2006–2019

### Towards genome-wide SNP genotyping

In 2005, Illumina introduced a novel SNP genotyping system, the BeadStation, for microarray-based SNP genotyping with ‘BeadChips’. In the same year, the SNP platform received funding from WCN to acquire a BeadStation instrument, facilitating scaling-up and (semi)automation of SNP genotyping for platform users. During 2001 and 2007, the International HapMap consortium established a map of linked human nucleotide variations (haplotypes), which enabled GWAS (11). The SNP Technology Platform upgraded its genotyping instrument in 2007 with the Infinium assay for genotyping and an iScan instrument, along with software for data interpretation. There was great interest among researchers in Sweden and in other countries to perform GWAS of human complex diseases in clinical sample cohorts with support by the SNP platform. In addition to SNP genotyping in GWAS, the SNP platform offered DNA Methylation using BeadChips with 450,000 CpG sites and later with 850,000 CpG sites. The number of analyzed samples processed at the SNP Platform grew rapidly, from a few hundred samples per year in 2005 to 30,000 samples per year in 2007. The genotyping volume reached a steady state at about 30,000 samples per year and has remained at this level for more than a decade.

### Next generation sequencing

In 2007, a paradigm shift for genome sequencing took place when the ‘Solexa’ sequencing technology was launched. The chemistry and technology for the Solexa system were developed during the period 1997–2006 by Shankar Balasubramanian and David Klenerman at Solexa Ltd, based at the University of Cambridge in the UK. The principle behind sequencing-by-synthesis involved several key steps: immobilization of double-stranded DNA templates and primers on a solid support, bridge amplification to generate clusters of single stranded DNA templates and primers, annealing of primers, and stepwise incorporation of fluorescently labeled, reversibly terminating nucleotides by a DNA polymerase and detection of the incorporated nucleotides, followed by cyclic repetitions of single base primer extension. In 2007, Solexa was acquired by Illumina Inc., and the Solexa/Illumina instrument became the first-generation Genome Analyzer for NGS. The capacity of this instrument was 1Gb (gigabase) of sequence data per run. In 2007, the SNP Technology Platform obtained the first Genome Analyzer in Sweden. It initially took some time to generate sequences from the first instrument, but fortunately, the Genome Analyzer was soon upgraded to a new version, and we learned to handle the sequence data. In 2007, after acquisition of the Genome Analyzer, the name of the SNP Technology Platform was changed to the SNP&SEQ Technology Platform. The Genome Analyzer was then upgraded about every 3 years with new Illumina HiSeq machines, with significantly higher capacity, lower costs, and better performance in each upgraded version.

In 2014, the SNP&SEQ Technology Platform and my research group moved from Uppsala University Hospital to the SciLifeLab premises at the Uppsala Biomedical Centre (BMC). One reason for the move was the lack of space at the hospital, and another that the SNP&SEQ Technology Platform had become part of SciLifeLab in 2013. The platform moved into excellent laboratories with sufficient space for large equipment and newly renovated office spaces. In 2015, the SNP&SEQ Technology Platform obtained five HiSeqX machines earmarked for human WGS, purchased jointly with five additional HiSeqX machines at the SciLifeLab node in Stockholm. These machines were used until 2019. In 2017, the SNP&SEQ Technology Platform obtained two NovaSeq 6,000 sequencers, with a sequencing output of up to 6 Tb (terabases) of sequence in less than 2 days. In 2024, an even larger NovaSeq X plus system is installed.

Since 2019, the SNP&SEQ Technology Platform is managed by Jessica Nordlund as a Director and Ulrika Liljedahl as a Head of Unit. Today, in 2024, the SNP&SEQ Platform has a staff of 34 persons, with core funding from the Swedish Research Council and SciLifeLab. The platform supports over 200 users projects and contributes to >100 publications annually. Although the current main sequencing applications at the SNP&SEQ-platform are SNP genotyping, RNA-sequencing, WGS, targeted sequencing, epigenetic analyses, and single-cell analysis, there is a growing interest for using sequencing as an output for other omics data types. One example, and a fast growing application area, is the innovation by the Uppsala-based company Olink Proteomics that protein abundance can be detected by coupling the proximity extension assay to a NGS read-out, enabling high throughput proteomics measurements (77).

## Concluding remarks

I have been fortunate to be part of the developing of genomics during almost four decades, from early microbial DNA diagnostics to high-precision disease genomics and epigenomics. My research work has been my main interest both in Helsinki and Uppsala. In Helsinki, during 1983–1990, in the Orion Gene Technology Laboratory, we pioneered innovative methods for diagnostics, which was a very exciting time period for me. During 1990–1998 in the Laboratory of Human Molecular Genetics at the National Public Health Institute as a senior scientist, I learned human genetics and obtained good friends among the group members while instructing them how to perform contamination-free PCRs. At Uppsala University, during 2000–2018, I supervised 19 highly motivated PhD students and 10 postdoctoral researchers. I have particularly enjoyed holding lab meetings with the members of the Molecular Medicine group and our collaborators. I am pleased to note that each one of the PhD students have good job positions in academia, biotech companies, or health care after obtaining their PhD degrees. I retired from work formally in 2017 but continued my research as Senior Professor at Uppsala University until 2022. I note that the prospects of treating ALL have increased greatly since WGS became feasible and has been implemented in routine clinical diagnostics at all major hospitals in Sweden. The treatment of the SLE disease may also be improved by recently developed antibody therapeutics in the USA. I believe that technology development has contributed significantly to the progress.
